# Prosthesis–patient mismatch after mitral valve replacement: A pooled meta‐analysis of Kaplan–Meier‐derived individual patient data

**DOI:** 10.1111/jocs.15108

**Published:** 2020-10-21

**Authors:** Anton Tomšič, Bardia Arabkhani, Jan W. Schoones, Jonathan R. G. Etnel, Nina A. Marsan, Robert J. M. Klautz, Meindert Palmen

**Affiliations:** ^1^ Department of Cardiothoracic Surgery Leiden University Medical Centre Leiden The Netherlands; ^2^ Walaeus Library Leiden University Medical Centre Leiden The Netherlands; ^3^ Department of Cardiothoracic Surgery Erasmus University Medical Centre Rotterdam The Netherlands; ^4^ Department of Cardiology Leiden University Medical Centre Leiden The Netherlands

**Keywords:** heart valve disease, mitral valve replacement, prosthesis–patient mismatch

## Abstract

**Objective:**

The hemodynamic effect and early and late survival impact of prosthesis–patient mismatch (PPM) after mitral valve replacement remains insufficiently explored.

**Methods:**

Pubmed, Embase, Web of Science, and Cochrane Library databases were searched for English language original publications. The search yielded 791 potentially relevant studies. The final review and analysis included 19 studies compromising 11,675 patients.

**Results:**

Prosthetic effective orifice area was calculated with the continuity equation method in 7 (37%), pressure half‐time method in 2 (10%), and partially or fully obtained from referenced values in 10 (53%) studies. Risk factors for PPM included gender (male), diabetes mellitus, chronic renal disease, and the use of bioprostheses. When pooling unadjusted data, PPM was associated with higher perioperative (odds ratio [OR]: 1.66; 95% confidence interval [CI]: 1.32–2.10; *p* < .001) and late mortality (hazard ratio [HR]: 1.46; 95% CI: 1.21–1.77; *p* < .001). Moreover, PPM was associated with higher late mortality when Cox proportional‐hazards regression (HR: 1.97; 95% CI: 1.57–2.47; *p* < .001) and propensity score (HR: 1.99; 95% CI: 1.34–2.95; *p* < .001) adjusted data were pooled. Contrarily, moderate (HR: 1.01; 95% CI: 0.84–1.22; *p* = .88) or severe (HR: 1.19; 95% CI: 0.89–1.58; *p* = .24) PPM were not related to higher late mortality when adjusted data were pooled individually. PPM was associated with higher systolic pulmonary pressures (mean difference: 7.88 mmHg; 95% CI: 4.72–11.05; *p* < .001) and less pulmonary hypertension regression (OR: 5.78; 95% CI: 3.33–10.05; *p* < .001) late after surgery.

**Conclusions:**

Mitral valve PPM is associated with higher postoperative pulmonary artery pressure and might impair perioperative and overall survival. The relation should be further assessed in properly designed studies.

## 1 INTRODUCTION

Prosthesis–patient mismatch (PPM) has been intensively studied in patients after aortic valve replacement.^1^, ^2^ In contrast, the hemodynamic and clinical consequences of PPM following mitral valve replacement (MVR) are less well established.

PPM after valve replacement occurs due to a mismatch in the prosthetic valve effective orifice area (EOA) in relation to the patient's body size, which is being used as an approximation of the patient's cardiac output. MVR remains a common procedure and contemporary data from the Society of Thoracic Surgery database demonstrate that MVR is performed in more than 40% of patients undergoing MV surgery in North America.^3^ The clinical consequences of PPM after MVR remain unclear as contradicting results, with some studies showing impaired outcomes in the presence of PPM^4^, ^5^ while others have failed to do so,^6^, ^7^ have been published to date. A number of the available studies was insufficiently powered to detect a clinically relevant effect and this could explain the lack of consistency in the available literature. Moreover, the influence of relevant methodological aspects (e.g., method of EOA calculation) on the results in the literature remains unexplored.

In an attempt to further explore the hemodynamic effect as well as the impact on early and late survival of PPM after MVR, a systematic review and meta‐analysis were performed.

## 2 METHODS

A systematic literature search of Pubmed, Embase, Web of Science, and Cochrane library was conducted by a biomedical information specialist. The detailed search strategy is described in Supporting Information Data A. Only full‐length studies in English were eligible for inclusion in the review. Two reviewers (A. T. and B. A.) independently assess the titles and abstracts of studies for eligibility. The Newcastle‐Ottawa Quality Assessment Scale was used to assess the quality of included studies. This systematic review and meta‐analysis were performed according to the Preferred Reporting Items for Systematic Reviews and Meta‐Analyses (PRISMA) guidelines (Supporting InformationMaterial H).^8^

### 2.1 Inclusion criteria

The following inclusion criteria were used: the publication was an original full‐article contribution in a peer‐reviewed journal; patients were adults; patients had undergone MVR with either a mechanical or bioprosthetic valve; ≥50 patients were included; PPM was assessed; patients were stratified in PPM and no‐PPM groups. In case of uncertainty, articles in full‐text were further evaluated. The reference lists of relevant studies were searched to identify any other full‐text article relevant to the review topic.

Studies that reported results of a “PPM” versus “no‐PPM” group were included in the “any PPM” pooled analyses. Studies that reported results for moderate and severe PPM were separately included in “moderate PPM” and “severe PPM” pooled analyses. For articles providing the results of both any PPM as well as moderate PPM or severe PPM subgroups on the endpoints of interest, the available data were included in both “any PPM” as well as “moderate PPM” and “severe PPM” pooled analyses.

### 2.2 Data extraction

From each study, either possibly related to the development of PPM or presenting a possible consequence of PPM, the following data were extracted: study design, number of patients, baseline characteristics, method of EOA determination, indexed EOA cut‐off threshold for PPM, and the number of patients with PPM. The following baseline characteristics were documented: patient age at operation, gender, presence of systemic and pulmonary hypertension (PH), diabetes mellitus, chronic renal disease, atrial fibrillation, impaired left ventricular function (as defined by the authors), and prosthesis type (biological or mechanical). In addition to early and late all‐cause mortality, data on echocardiographic parameters possibly related to PPM were recorded. Microsoft Excel (Microsoft Corp.) was used to extract data.

### 2.3 Study endpoints

Primary endpoints were perioperative mortality and overall survival. Secondary outcomes included residual PH (defined as the absence of postoperative pulmonary artery pressure normalization, in particular, residual pulmonary artery pressure >40 mmHg, as defined in the studies included in the review) and postoperative systolic pulmonary artery pressure. Based on the timing of echocardiographic measurement, studies were stratified in early (echocardiographic assessment during the index hospitalization) and late (echocardiographic assessment at a later time point during patient follow‐up) period.

### 2.4 Statistical analysis

Meta‐analyses were performed using Review Manager, Version 5.3 (Copenhagen: The Nordic Cochrane Centre, The Cochrane Collaboration, 2014). Fixed and random‐effects models were used to obtain pooled estimates. For late mortality, study results were subgrouped by study design type: unmatched/unadjusted observational data, risk‐adjusted observational data, and propensity score‐matched data. Studies that reported both matched or risk‐adjusted and unmatched/unadjusted data were included separately for subgroup comparisons. Heterogeneity was examined with the *I* ^2^ statistics. The degree of heterogeneity was graded as low (*I* ^2^ < 25%), moderate (*I* ^2^ = 25%–75%), and high (*I* ^2^ > 75%). Sources of heterogeneity were explored by subgroup analyses of study (method used to obtain EOA, study location, year of publication) or patient characteristics (patient age). Additionally, a meta‐regression was performed to assess the potential effect of clinically relevant modulating factors (including patient age, gender, atrial fibrillation, hypertension impaired left ventricular ejection fraction [LVEF], and diabetes mellitus) on overall survival. Funnel plots were produced for visualization of possible bias. Meta‐analyses results are displayed in forest plots. *p* < .05 was considered statistically significant.

Late mortality was extracted as a hazard ratio (HR) with corresponding variance. For studies that did not report this, a logarithmic HR with corresponding variance was estimated from the published Kaplan–Meier curves for survival for the PPM and no‐PPM groups separately. Published Kaplan–Meier curves were digitized and an estimate of the individual patient time‐to‐event data was then extrapolated from the digitized curve coordinates, assuming a constant rate of censorship between each time point at which the number of patients at risk was specified.^9^ Published Kaplan–Meier curves were digitized using Engauge Digitizer (version 10.3, http://markummitchell.github.io/engauge-digitizer). Extrapolation of estimated individual patient time‐to‐event data from the digitized curves was performed in R statistical software (version 3.3.2, R Development Core Team; R Foundation for Statistical Computing).

## 3 RESULTS

The database search yielded 791 potentially relevant studies (Supporting Information Data B). After removal of duplicates and title‐abstract screening, 25 full‐text original articles were reviewed in further detail. Four studies were additionally excluded due to no differentiation in PPM and no‐PPM groups in two, use of geometric orifice area to assess PPM in one and an insufficient number of patients included in one. Sixteen retrospective single‐center studies,^4^, ^5^, ^6^, ^10^, ^11^, ^12^, ^13^, ^14^, ^15^, ^16^, ^17^, ^18^, ^19^, ^20^, ^21^, ^22^ two retrospective multicenter studies,^7^, ^23^ and one prospective study^24^ were included in the final review and meta‐analysis. Two studies identified were meta‐analyses.^25^, ^26^

In one study,^19^ a two‐tailed analysis was performed and EOA was obtained from referenced values or measured with the continuity equation (CE) method. In another study,^15^ a three‐tailed analysis was performed and EOA was obtained by using either referenced values or measured with either the CE or pressure half‐time (PHT) method. Only data derived from the analysis based on the EOA measured with the CE method were included. Results of the study quality assessment are presented in Supporting Information Data C.

Nineteen studies with a total of 11,675 patients were included in the meta‐analysis. The baseline characteristics of all patients included are presented in Supporting Information Data D. The 1.2‐cm^2^/m^2^ cut‐off threshold was used to define any relevant PPM in the majority of studies (Table 1). Eight studies (including 5887 patients) divided the PPM group into moderate and severe PPM subgroups. The 0.9‐cm^2^/m^2^ cut‐off threshold was used to define severe PPM in all of these studies. Overall, the prevalence of any PPM was 50%. In the eight studies providing data on the severity of PPM, moderate PPM was seen in 57% and severe PPM in 13%.

**Table 1:** Study characteristics

					Type of prosthesis		Indexed EOA cut‐off (cm^2^/m^2^)			Prosthesis–patient mismatch^c^		
Study	Study location	Inclusion period	Study design	No. of patients	Mechanical prosthesis	Biological prosthesis	Any (moderate)	Severe	EOA measurement	Any	Moderate	Severe
Akuffu et al.^22^	China	2013–2015	Retrospective	1067	868 (81)	199 (19)	≤1.2	‐	Referenced	189 (18)	‐	‐
Ammannaya et al.^10^	India	1990–2016	Retrospective	500	500 (100)	0 (0)	≤1.2	‐	Calculated CE	186 (36)	‐	‐
Angeloni et al.^11^	Italy	2004–2011	Retrospective	210	135 (64)	75 (36)	≤1.2	‐	Calculated CE	88 (42)	‐	‐
Aziz et al.^12^	USA	1992–2008	Retrospective	765	440 (58)	325 (42)	≤1.2	<0.9	Referenced	393 (51)	286 (37)	107 (14)
Borracci et al.^13^	Argentina	2009–2013	Retrospective	136	78 (57)	58 (43)	≤1.2	<0.9	Referenced	96 (71)	60 (44)	36 (26)
Bouchard et al.^5^	Canada	1992–2005	Retrospective	714	714 (100)	0 (0)	≤1.2; ≤1.3; ≤1.4	‐	Referenced	74 (10)	‐	‐
Cao et al.^23^	China (multicenter)	2000–2008	Retrospective	493	493 (100)	0 (0)	≤1.2	‐	Calculated CE	157 (32)	‐	‐
Cho et al.^15^	Korea	‐	Retrospective	166	129 (78)	37 (22)	≤1.2	≤0.9	Calculated CE^a^	103 (62)	80 (48)	23 (14)
El Midany et al.^24^	Egypt	2013–2017	Prospective	715	715 (100)	0 (0)	≤1.2	≤0.9	Calculated CE	382 (53)	287 (40)	95 (13)
Hwang et al.^4^	Korea	1992–2012	Retrospective	760	642 (84)	118 (16)	≤1.2	‐	Referenced	147 (19)	‐	‐
Jamieson et al.^14^	Canada	1982–2002	Retrospective	2440	1083 (44)	1357 (56)	≤1.2	≤0.9	Referenced	2095 (86)	1696 (70)	399 (16)
Lam et al.^16^	Canada	1985–2005	Retrospective	884	657 (74)	227 (26)	≤1.25	‐	Referenced (or provided by manufacturer)	280 (32)	‐	‐
Lee et al.^6^	Korea	2000–2013	Retrospective	445	361 (81)	84 (19)	≤1.2	≤0.9	Calculated CE	165 (37)	157 (35)	8 (2)
Li et al.^17^	Canada	2003–2003	Retrospective	56	47 (84)	9 (16)	≤1.2	‐	Calculated CE	40 (71)	‐	‐
Magne et al.^18^	Canada	1986–2005	Retrospective	929	789 (85)	140 (15)	≤1.2	≤0.9	Referenced or calculated CE	725 (78)	644 (69)	81 (9)
Matsuura et al.^19^	Japan	1995–2008	Retrospective	163	112 (69)	51 (31)	≤1.2	‐	Calculated pressure half‐time method^b^	17 (10)	‐	‐
Sakamoto et al.^20^	Japan	1992–2005	Retrospective	84	75 (89)	9 (11)	≤1.2	‐	Calculated pressure half‐time method	25 (30)	‐	‐
Sato et al.^21^	Japan	2000–2011	Retrospective	142	110 (77)	32 (23)	≤1.2	‐	Referenced or calculated CE	60 (42)	‐	‐
Shi et al.^7^	Australia (multicenter)	2001–2009	Retrospective	1006	622 (62)	384 (38)	≤1.2	≤0.9	Referenced	665 (66)	532 (53)	133 (13)
			Total	11,675 (100)	8570 (73)	3105 (27)			5887 (50)	3742/6602 (57)	882/6602 (13)

*Note*: Data are presented as *N* (%).

Abbreviations: CE, continuity equation; EOA, effective orifice area; PPM, prosthesis–patient mismatch.

^a^ The article provided a comparison of the effect of different methods of EOA measurement; only data derived from the continuity equation method were included.

^b^ In the article, EOA is derived from two methods; only data derived from the pressure half‐time method were included.

^c^ For articles providing both the results of any PPM as well as moderate or severe PPM subgroups on the endpoints of interest, the available data were included in both “any PPM” as well as “moderate PPM” and “severe PPM” pooled analyses.

The EOA was measured in vivo with the CE method in all participants in 7 (37%) studies. The PHT method was used to measure the EOA in 2 (10%) studies. Other studies used either referenced values from the literature or provided by the manufacturer (*n* = 7; 37%) or a combination of referenced values and in vivo measurements (*n* = 3; 16%).

### 3.1 Risk factors for PPM

The use of bioprostheses demonstrated the strongest correlation with PPM (Supporting Information Data E). Furthermore, hypertension, PH, diabetes mellitus, and chronic renal disease were all associated with PPM. In contrast, female gender was related to a lower risk of PPM. Similar results were found when the risk factors for moderate or severe PPM were explored individually. The use of bioprostheses, diabetes mellitus, and impaired left ventricular function were associated with an increased risk of moderate PPM while female gender and atrial fibrillation were associated with a lower risk of moderate PPM. Similarly, the use of bioprostheses, diabetes mellitus, and chronic renal disease was associated with an increased risk of severe PPM.

### 3.2 Perioperative mortality

**Figure 1:**
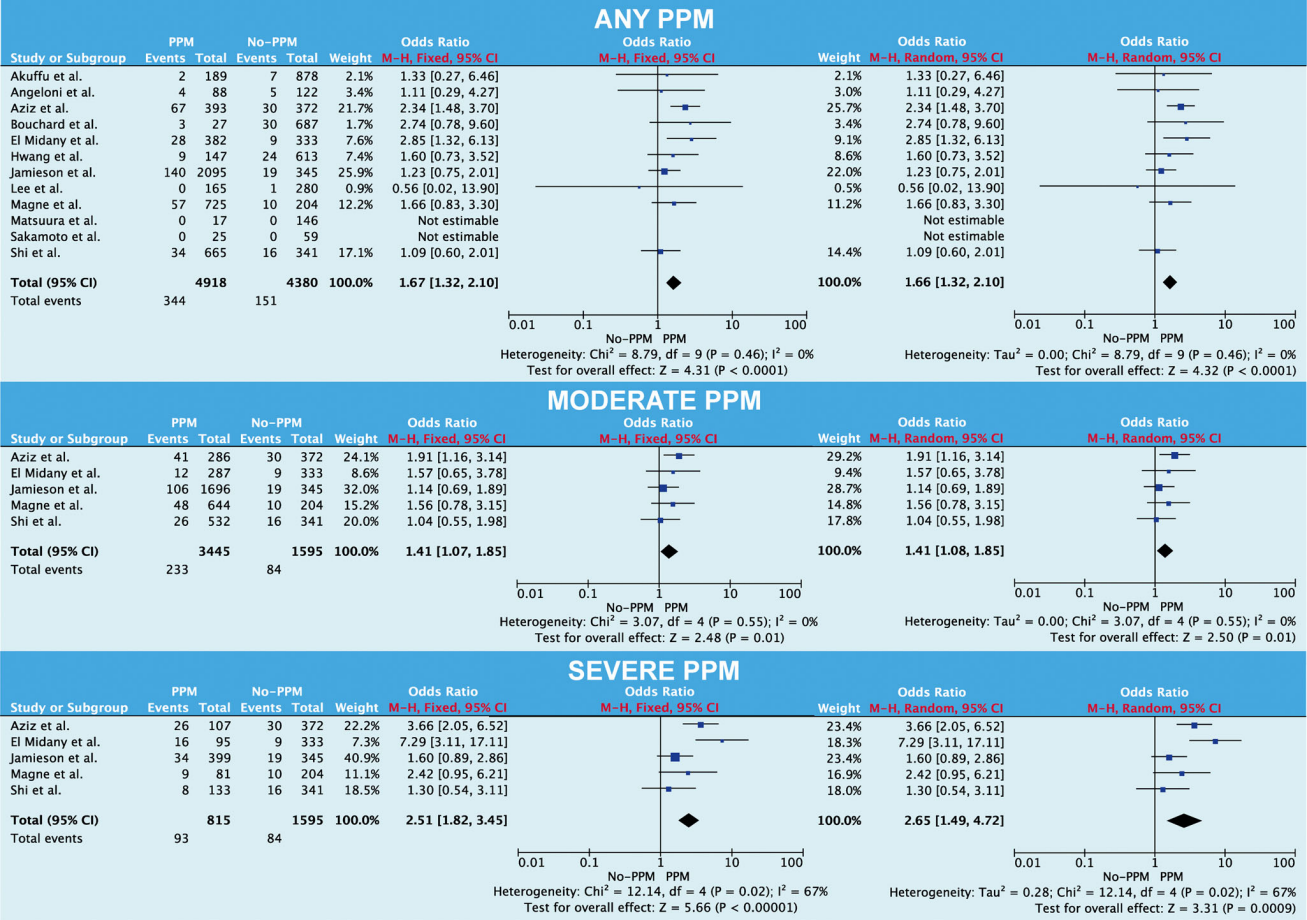
Forest plot analysis on the effect of prosthesis–patient mismatch (PPM) on perioperative mortality for the following: (top) any degree of PPM versus no PPM; (middle) moderate PPM versus no PPM; (bottom) severe PPM versus no PPM

Any PPM was associated with increased perioperative mortality (odds ratio [OR]: 1.66; 95% confidence interval [CI]: 1.32–2.10; *p* < .001; Figure 1) and no asymmetry was observed on funnel plot analysis (Supporting Information Data F). Similar findings were seen when moderate PPM (OR: 1.41; 95% CI: 1.08–1.85; *p* = .01) and severe PPM (OR: 2.65; 95% CI: 1.49–4.72; *p* < .001) were compared with no‐PPM separately. Subgroup analysis (Supporting Information Data G) revealed no significant heterogeneity.

### 3.3 Overall survival

**Figure 2:**
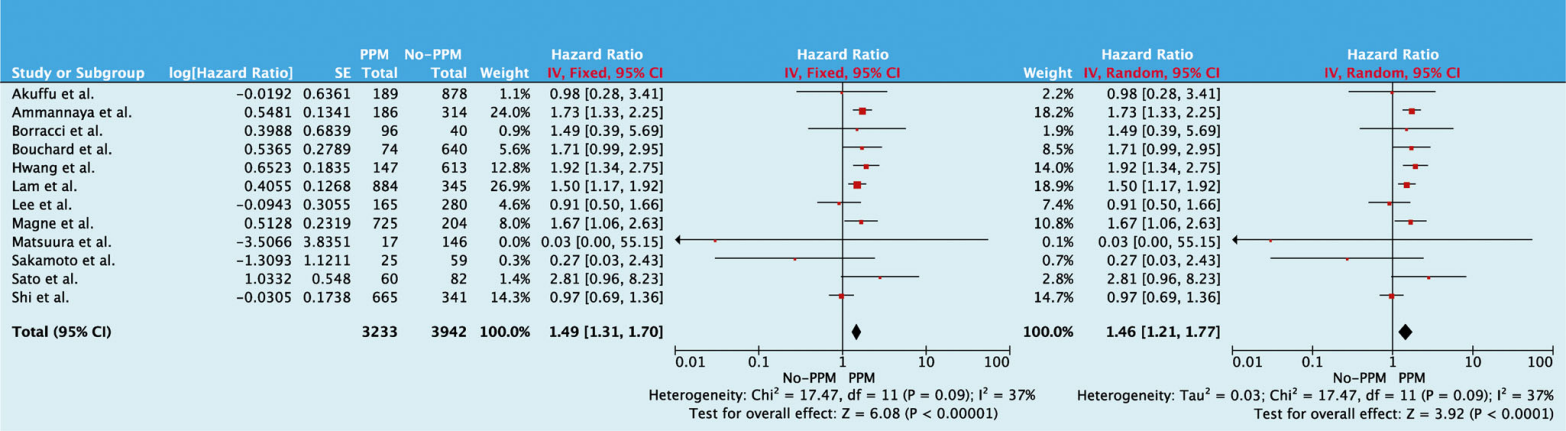
Forest plot analysis on the effect of any degree of prosthesis–patient mismatch (PPM) on overall survival

PPM was associated with higher overall mortality when compared to patients without PPM when unadjusted observational data were pooled (HR: 1.46; 95% CI: 1.21–1.77; *p* < .001; Figure 2). Funnel plot analysis revealed asymmetry and we repeated the analysis while excluding studies in which the EOA was measured by the PHT method (Supporting Information Data H). PPM remained associated with decreased overall survival (HR: 1.49; 95% CI: 1.24–1.78; *p* < .001) and no asymmetry was seen on funnel plot analysis. Subgroup analysis (Supporting Information Data G) revealed no significant heterogeneity.

**Figure 3:**
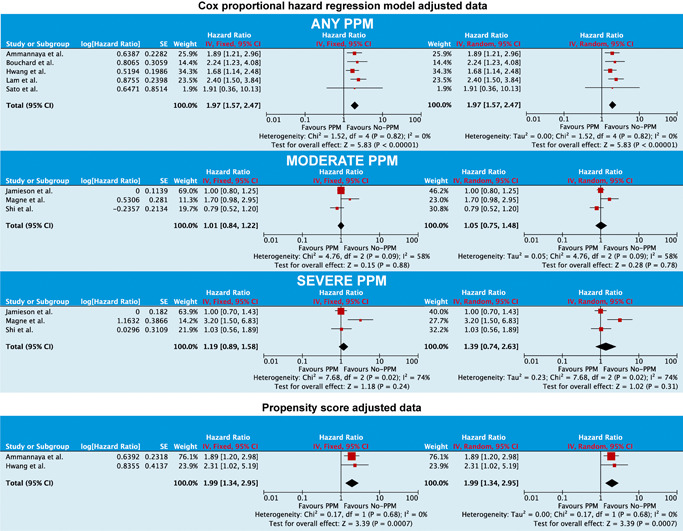
Forest plot analysis on the effect of prosthesis–patient mismatch (PPM) on overall survival when Cox proportional‐hazards model (top) and propensity score‐matched data (bottom) were pooled

When adjusted observational data were pooled (Figure 3), PPM was associated with poorer overall survival (HR: 1.97; 95% CI: 1.57–2.47; *p* < .001). In contrast, moderate PPM (HR: 1.05; 95% CI: 0.75–1.48; *p* = .78) and severe PPM (HR: 1.39, 95% CI: 0.74–2.63, *p* = .31) were not related to poorer survival when these were compared with no‐PPM separately. Pooled propensity score‐matched data (Figure 3) revealed poorer overall survival with PPM (HR: 1.99; 95% CI: 1.34–2.95; *p* < .001).

### 3.4 Secondary outcomes

PPM was associated with higher pulmonary pressure both in the early (mean difference: 8.88 mmHg; 95% CI: 3.03–14.73; *p* = .003) as well as in the late (mean difference: 7.88 mmHg; 95% CI: 4.72–11.05; *p* < .001) postoperative phase (Supporting Information Data I). When the effect of PPM on pulmonary pressure was explored by means of the incidence of residual PH, no effect of PPM was seen in the early (OR: 3.00; 95% CI: 0.42–21.52; *p* = .28) while a negative effect was seen in the late (OR: 5.78; 95% CI: 3.33–10.05; *p* < .001) postoperative phase.

### 3.5 Meta‐regression analysis

Univariable meta‐regression analysis (Supporting Information Data J) demonstrated interaction between hypertension (B‐coefficient: −0.013; standard error: 0.005; *p* = .041), impaired left ventricular function (B‐coefficient: −0.017; standard error: 0.002; *p* = .002) and female gender (B‐coefficient: 0.23; standard error: 0.007; *p* = .005) and overall survival.

## 4 DISCUSSION

The most important finding of our study is that PPM resulted in reduced perioperative and overall survival. The results, however, need to be interpreted with caution as the method of EOA determination varied significantly across studies and the majority of data originate from unadjusted observational data.

### 4.1 Method of EOA determination

In a recent study, Cho et al.^15^ explored the effect of the method of EOA determination on the incidence and hemodynamic consequences of PPM after MVR. Remarkable differences were observed as the incidence of PPM ranged from 7% when measured with the PHT method to 49% and 62% when obtained from referenced values or measured with the CE method, respectively. An association between PPM and pulmonary artery pressure was seen only when the EOA was measured with the CE method. Dumesnil et al.^27^ similarly reported that the PHT method overestimates the EOA when compared with the CE method and the use of the PHT has been discouraged in a recent recommendation by the European Association of Cardiovascular Imaging.^28^ For clinical and study purposes, the CE method should thus be encouraged.

### 4.2 Risk factors for PPM

The use of bioprostheses rather than mechanical prostheses demonstrated the strongest correlation with PPM development. This is in line with the findings of studies on the risk factors associated with the development of PPM after aortic valve replacement^1^, ^2^ and is presumably related to the relatively smaller EOA of bioprostheses in relation to the geometric orifice area. Other patient characteristics identified as risk factors for the development of PPM are likely related to the impact these are to have on mitral valve circumference or relation to patient body surface area.

The identification of bioprostheses as a prominent risk factor for the development of PPM somehow challenges the recent trend of lowering the age margin for MVR with a bioprosthesis.^29^, ^30^ Nevertheless, the use of mechanical prostheses does not eliminate the risk for PPM development and other clinical factors (e.g., use of oral anticoagulation) likely play a more prominent role in determining patient survival and quality of life. For anatomical reasons, a mechanical prosthesis could be favored over a biological one in carefully selected patients to lower the possibility of PPM development.

### 4.3 Hemodynamic consequences of PPM

PPM following MVR resulted in higher pulmonary artery pressures. When tested as a binary variable, the presence of PPM resulted in an almost sixfold increase in the probability of residual PH. These findings provide theoretical grounds for a negative impact of PPM on clinical outcomes following MVR. This is supported by the study of Angeloni et al.,^11^ who observed that PPM will diminish right ventricular reverse remodeling and result in a higher incidence of functional tricuspid valve regurgitation.

### 4.4 Clinical impact of PPM

Perioperative mortality was higher in the presence of PPM. This could be due to residual pulmonary congestion that leads to prolonged mechanical ventilation and respiratory tract infections, as suggested by Hwang et al.^4^ However, the method of EOA determination could have an effect on this observation. The number of studies in which the EOA was individually measured by the recommended CE method was surprisingly low and only two studies including 655 patients were available for subanalysis. Late survival was also negatively affected by the presence of PPM. Again, these data need to be interpreted in line with the limitations of the studies available for review in mind. The number of studies in which the EOA was measured with the recommended CE method was limited to two with a total of 945 patients included.

The theoretical effect of PPM on both early and late mortality is driven by obstructed transprosthetic flow, reflected by elevated pulmonary artery pressures. This only holds true when PPM is measured with the CE method. As the majority of studies available for analysis did not use the CE method to measure the EOA, the presented results should be interpreted with caution. Supported by the demonstrated effect on postoperative PAH incidence, PPM can be seen as a factor that can potentially impair perioperative and late outcomes but further high‐quality studies are needed before clear conclusions can be drawn.

### 4.5 Comparison with previous studies

Two previous meta‐analyses have explored the effect of PPM after MVR.^25^, ^26^ However, certain methodological limitations of these studies need to be acknowledged as well as limitations regarding the interpretation of the results presented. Our meta‐analysis was the first to explore the risk factors related to the development of PPM after MVR, providing guidance for clinicians in identifying patients at risk. Moreover, we were able to extract the HRs of time‐related outcomes, providing a more accurate assessment of the consequences of PPM.

We have furthermore explored the effect that various methods of EAO determination have on the clinical outcomes related to PPM and warn against definite conclusions being drawn without taking these limitations into account. Furthermore, certain observations previously made (e.g., improved LVEF in the absence of PPM) seem to be more likely a consequence of chance than a relevant effect of PPM. We attempted to collect data on the preservation of the subvalvular apparatus during MVR, a possible explanation for decreased postoperative left ventricular function. No significant differences were seen; however, the number of studies reporting this variable was surprisingly low.

### 4.6 Clinical applicability of PPM after MVR

In the literature, PPM seems to be a well‐established concept and has, in the case of aortic valve replacement, been included in the guidelines that recommend transcatheter aortic valve implantation over surgical valve replacement when PPM is expected.^31^ Despite the fact that our results demonstrate an effect of PPM on postoperative pulmonary artery pressure and, possibly, early and late overall survival, it should be understood that PPM is a population‐based concept with several limitations and cannot be easily translated to individual patient level. PPM is calculated by indexing the prosthetic EOA to patient BSA that is assumed to adequately estimate patient cardiac output in an independent one‐to‐one linear relationship (cardiac output = constant × BSA).^32^ However, this is not true as a positive intercept is present in the relationship between BSA and cardiac output (cardiac output = constant × BSA + *N*). Consequently, the cardiac output/BSA ratio is greater for a lower than a higher BSA. It should also be acknowledged that other patient characteristics, for example, patient age, importantly influence cardiac output. It is, therefore, not surprising that PPM after aortic valve replacement has been shown to have a less profound clinical effect in older and obese patients in whom cardiac output is less than expected.^1^, ^33^ We could not explore the effect of these characteristics on patient outcomes in the case of MVR due to the lack of data available.

A single cut‐off value to define PPM by indexing the EOA to BSA, not taking into account the variability in the cardiac output/BSA ratio and irrespective of other characteristics influencing cardiac output, is thus misleading. Nevertheless, we reason that the results of our meta‐analysis do reflect the population‐based clinical effect of PPM after MVR. This is related to the fact that patients who are classified as having PPM based on indexing the EOA to BSA were also at higher risk of actually having PPM (EOA/cardiac output). The limitations of the PPM concept do limit the possibilities for accurate clinical decision‐making on individual patient basis but do support the population‐based effort to lower the burden of PPM. In the case of MVR, the possibilities seem less straightforward than in the case of aortic valve replacement but include the use of prostheses with the largest EOA/geometric orifice area ratio, especially in patients with small mitral valve annuli, future adjustments in prosthetic valve design and, as proposed by Angeloni et al.,^11^ a lower threshold for concomitant tricuspid valve repair. Moreover, efforts to implant the largest size prosthesis, including complete decalcification of a calcified mitral valve annulus, seem justified. Reliable preoperative identification of patients at risk of developing postoperative PPM would allow for further optimization of the decision‐making process of the type of prosthesis implanted.

## 5 LIMITATIONS

The most important limitation is the variety of methods of EOA measurement in the studies included in this review. As a high number of studies obtained the EOA from referenced values, a significant number of patients included in the review might have been inappropriately classified in the PPM or no‐PPM groups; this also holds true for studies in which the PHT method was used to measure the EOA. Moreover, we did not perform an individual data meta‐analysis but based our analyses on the data available in the literature. Nevertheless, our meta‐analysis presents the largest study on the effect of PPM after MVR performed to date. Based on the available literature, the definition of PPM as a categorical variable seems widely accepted. A transformation of a continuous variable into a categorical one is related to several limitations and future studies should explore the clinical validity of these cut‐off points. The results obtained should be seen as hypothesis‐generating. Lastly, the current study was not registered at PROSPERO international registry of systematic reviews.

## 6 CONCLUSIONS

Perioperative and late survival may be impaired by the presence of mitral valve PPM. This is possibly related to the presence of residual PH. Due to methodological limitations (method of EOA measurement) in the available literature, the results of our meta‐analysis should be regarded as hypothesis‐generating and further studies should establish the applicability of our results on individual patient basis.

## CONFLICT OF INTERESTS

The authors declare that there are no conflict of interests.

## Supporting information

Supporting information.
