# Supplementary material for: Prosthesis–patient mismatch after mitral valve replacement: A pooled meta‐analysis of Kaplan–Meier‐derived individual patient data
Source: J Card Surg. 2020 Oct 21;35(12):3477–85. doi: 10.1111/jocs.15108 (PMC7756724; doi:10.1111/jocs.15108)
Supplement: Supplementary file 1 — Supporting information. [file JOCS-35-3477-s001.docx]

**Supplementary data A.** Detailed search strategy.

**PubMed**

((("patient prosthesis mismatch"[tw] OR "patient prosthetic mismatch"[tw] OR "prothesis patient mismatch"[tw] OR "PPM"[tw] OR "Prosthesis Fitting"[mesh] OR "prosthesis fitting"[tw]) AND ("mitral valve replacement"[tw] OR "MVR"[tw] OR "mitral valve prostheses"[tw] OR "mitral valve prosthesis"[tw] OR "mitral valve prosthetic"[tw] OR "mitral valve prosthetics"[tw] OR (("Mitral Valve"[Mesh] OR "mitral valve"[tw] OR "mitral valves"[tw] OR "bicuspid valve"[tw] OR "bicuspid valves"[tw]) AND ("Heart Valve Prosthesis Implantation"[Mesh] OR "Heart Valve Prosthesis"[mesh] OR "replacement"[tw] OR replac*[tw] OR prosthe*[tw])))) OR (("mismatch"[tw] OR mismatch*[tw]) AND ("mitral valve replacement"[tw] OR "MVR"[tw] OR "mitral valve prostheses"[tw] OR "mitral valve prosthesis"[tw] OR "mitral valve prosthetic"[tw] OR "mitral valve prosthetics"[tw] OR (("Mitral Valve"[Mesh] OR "mitral valve"[tw] OR "mitral valves"[tw] OR "bicuspid valve"[tw] OR "bicuspid valves"[tw]) AND ("Heart Valve Prosthesis Implantation"[Mesh] OR "replacement"[tw] OR replac*[tw] OR prosthe*[tw])))) OR (("mismatch"[tw] OR mismatch*[tw]) AND ("mitral valve"[tw] OR "mitral valves"[tw] OR "Mitral Valve"[Mesh] OR "Mitral Valve Stenosis"[Mesh] OR "Mitral Valve Prolapse"[Mesh] OR "Mitral Valve Insufficiency"[Mesh])) OR (("mismatch"[tw] OR mismatch*[tw]) AND ("mitral"[tw] OR mitra*[tw]))) AND english[la] NOT ("Animals"[mesh] NOT "Humans"[mesh])

**Embase**

((("patient prosthesis mismatch".mp OR "patient prosthetic mismatch".mp OR "prothesis patient mismatch".mp OR "PPM".mp OR "Prosthetic Fitting"/ OR "prosthesis fitting".mp) AND ("mitral valve replacement"/ OR "mitral valve replacement".mp OR "MVR".mp OR "mitral valve prostheses".mp OR "mitral valve prosthesis".mp OR "mitral valve prosthetic".mp OR "mitral valve prosthetics".mp OR (("Mitral Valve"/ OR "mitral valve".mp OR "mitral valves".mp OR "bicuspid valve".mp OR "bicuspid valves".mp) AND ("Heart Valve Replacement"/ OR "replacement".mp OR replac*.mp OR prosthe*.mp)))) OR (("mismatch".mp OR mismatch*.mp) AND ("mitral valve replacement"/ OR "mitral valve replacement".mp OR "MVR".mp OR "mitral valve prostheses".mp OR "mitral valve prosthesis".mp OR "mitral valve prosthetic".mp OR "mitral valve prosthetics".mp OR (("Mitral Valve"/ OR "mitral valve".mp OR "mitral valves".mp OR "bicuspid valve".mp OR "bicuspid valves".mp) AND ("Heart Valve Replacement"/ OR "replacement".mp OR replac*.mp OR prosthe*.mp)))) OR (("mismatch".mp OR mismatch*.mp) AND ("mitral valve".mp OR "mitral valves".mp OR "Mitral Valve"/ OR "Mitral Valve Stenosis"/ OR "Mitral Valve Prolapse"/ OR "Mitral Valve Insufficiency"/)) OR (("mismatch".mp OR mismatch*.mp) AND ("mitral".mp OR mitra*.mp))) AND english.la AND exp "Humans"/ NOT (conference review or conference abstract).pt

**Web of Science**

TS=((("patient prosthesis mismatch" OR "patient prosthetic mismatch" OR "prothesis patient mismatch" OR "PPM" OR "Prosthetic Fitting" OR "prosthesis fitting") AND ("mitral valve replacement" OR "mitral valve replacement" OR "MVR" OR "mitral valve prostheses" OR "mitral valve prosthesis" OR "mitral valve prosthetic" OR "mitral valve prosthetics" OR (("Mitral Valve" OR "mitral valve" OR "mitral valves" OR "bicuspid valve" OR "bicuspid valves") AND ("Heart Valve Replacement" OR "replacement" OR replac* OR prosthe*)))) OR (("mismatch" OR mismatch*) AND ("mitral valve replacement" OR "mitral valve replacement" OR "MVR" OR "mitral valve prostheses" OR "mitral valve prosthesis" OR "mitral valve prosthetic" OR "mitral valve prosthetics" OR (("Mitral Valve" OR "mitral valve" OR "mitral valves" OR "bicuspid valve" OR "bicuspid valves") AND ("Heart Valve Replacement" OR "replacement" OR replac* OR prosthe*)))) OR (("mismatch" OR mismatch*) AND ("mitral valve" OR "mitral valves" OR "Mitral Valve" OR "Mitral Valve Stenosis" OR "Mitral Valve Prolapse" OR "Mitral Valve Insufficiency")) OR (("mismatch" OR mismatch*) AND ("mitral" OR mitra*))) AND la=english NOT ti=(veterinary OR rabbit OR rabbits OR animal OR animals OR mouse OR mice OR rodent OR rodents OR rat OR rats OR pig OR pigs OR porcine OR horse* OR equine OR cow OR cows OR bovine OR goat OR goats OR sheep OR ovine OR canine OR dog OR dogs OR feline OR cat OR cats)

**Cochrane library**

((("patient prosthesis mismatch" OR "patient prosthetic mismatch" OR "prothesis patient mismatch" OR "PPM" OR "Prosthetic Fitting" OR "prosthesis fitting") AND ("mitral valve replacement" OR "mitral valve replacement" OR "MVR" OR "mitral valve prostheses" OR "mitral valve prosthesis" OR "mitral valve prosthetic" OR "mitral valve prosthetics" OR (("Mitral Valve" OR "mitral valve" OR "mitral valves" OR "bicuspid valve" OR "bicuspid valves") AND ("Heart Valve Replacement" OR "replacement" OR replac* OR prosthe*)))) OR (("mismatch" OR mismatch*) AND ("mitral valve replacement" OR "mitral valve replacement" OR "MVR" OR "mitral valve prostheses" OR "mitral valve prosthesis" OR "mitral valve prosthetic" OR "mitral valve prosthetics" OR (("Mitral Valve" OR "mitral valve" OR "mitral valves" OR "bicuspid valve" OR "bicuspid valves") AND ("Heart Valve Replacement" OR "replacement" OR replac* OR prosthe*)))) OR (("mismatch" OR mismatch*) AND ("mitral valve" OR "mitral valves" OR "Mitral Valve" OR "Mitral Valve Stenosis" OR "Mitral Valve Prolapse" OR "Mitral Valve Insufficiency")) OR (("mismatch" OR mismatch*) AND ("mitral" OR mitra*))) AND la=English

The search was performed on August 15^st^ 2019. In total, 791 articles were identified. All search results were imported into EndNote X7. Duplicate search results were removed and the remaining 435 results were filtered; abstracts, letters, editorials, and case-reports were eliminated. Results published in non-peer reviewed journals or not meeting the predefined inclusion criteria were eliminated. No contact with authors was made.

**Supplementary data B.** PRISMA flow diagram.

**Supplementary data C.** Quality assessment of the included observational studies using the Newcastle-Ottawa Scale. Quality assessment was based on the primary outcome of the study reviewed.

| Study | Quality assessment criteria | | | | | | | | | Overall quality score |
| --- | --- | --- | --- | --- | --- | --- | --- | --- | --- | --- |
|  | Selection | | | | Comparability | | Outcome | | |  |
|  | Representativeness of exposed cohort | Selection of the non-exposed cohort | Ascertainment of exposure | Demonstration that outcome of interest was not present at start of study | Comparability of study cohorts for most important factor (age and gender) | Comparability of study cohorts for comorbidities | Assessment of outcome | Was follow-up long enough for outcomes to occur | Adequacy of follow-up of cohorts |  |
| Akkuffu et al., 2018 | Yes | Yes | Yes | Yes | No | No | Yes | Yes (2.5 y) | No statement | 6 |
| Ammannaya et al., 2017 | Yes | Yes | Yes | Yes | Yes | Yes | Yes | Yes (8.15 y) | No statement | 8 |
| Angeloni et al., 2013 | Yes | Yes | Yes | Yes | No | No | Yes | Yes (2.3 y) | Yes (100%) | 7 |
| Aziz et al., 2010 | Yes | Yes | Yes | Yes | No | No | Yes | Yes (4.8 y) | Yes (100%) | 7 |
| Borracci et al., 2016 | Yes | Yes | Yes | Yes | No | No | Yes | Yes (3.1 y) | No statement | 6 |
| Bouchard et al., 2010 | Yes | Yes | Yes | Yes | Yes | Yes | Yes | No statement | No statement | 7 |
| El Midany et al., 2019 | Yes | Yes | Yes | Yes | No | No | Yes | Yes (2.2 y) | No (81.5%) | 6 |
| Hwang et al., 2016 | Yes | Yes | Yes | Yes | Yes | Yes | Yes | Yes (10.6 y) | Yes (96.4%) | 9 |
| Jamieson et al., 2009 | Yes | Yes | Yes | Yes | Yes | Yes | Yes | Yes (6.1 y) | Yes (97.8%) | 9 |
| Lam et al., 2007 | Yes | Yes | Yes | Yes | Yes | Yes | Yes | Yes (5.1 y) | Yes (100%) | 9 |
| Lee et al., 2017 | Yes | Yes | Yes | Yes | No | No | Yes | Yes (8.7 y) | No statement | 6 |
| Magne et al., 2007 | Yes | Yes | Yes | Yes | Yes | Yes | Yes | Yes (6.3 y) | No statement | 8 |
| Matsuura et al., 2011 | Yes | Yes | Yes | Yes | No | No | Yes | Yes (4.8 y) | No statement | 6 |
| Sakamoto et al., 2010 | Yes | Yes | Yes | Yes | No | No | Yes | Yes (8.5 y) | Yes (96%) | 7 |
| Sato et al., 2014 | Yes | Yes | Yes | Yes | Yes | Yes | Yes | Yes (7.0 y) | Yes (96.5%) | 9 |
| Shi et al., 2011 | Yes | Yes | Yes | Yes | Yes | Yes | Yes | No statement | Yes (97.4%) | 8 |

**Supplementary data D.** Baseline characteristics.

|  | No-PPM (5788) | PPM (5887) | P-value |
| --- | --- | --- | --- |
| Age (years) | 57.0±13.7 | 61.9±13.2 | <0.001 |
| Gender (female) | 3517/5417 (65) | 2752/5494 (50) | <0.001 |
| Body surface are (m2) | 1.61±0.22 (n=5066) | 1.79±0.25 (n=5753) | <0.001 |
| Body mass index (kg/m2) | 22.5±4.21 (n=1564) | 26.4±5.17 (n=2949) | <0.001 |
| Type of prosthesis implanted |  |  | <0.001 |
| Bioprosthesis | 799 (14) | 2307 (39) |  |
| Mechanical | 4989 (86) | 3580 (61) |  |
| Systemic hypertension | 650/3171 (20) | 889/2627 (34) | <0.001 |
| Pulmonary hypertension | 651/1087 (60) | 1323/2746 (48) | <0.001 |
| Atrial fibrillation | 1904/4145 (46) | 1817/5058 (36) | <0.001 |
| Impaired left ventricular function | 522/2198 (24) | 916/3098 (30) | <0.001 |
| Chronic renal disease | 90/1794 (5) | 253/2103 (12) | <0.001 |
| Diabetes mellitus | 356/3544 (10) | 535/3020 (18) | <0.001 |
| Type of mitral valve lesion |  |  | <0.001 |
| Stenosis | 601/1276 (47) | 624/1316 (47) |  |
| Regurgitation | 387/1276 (30) | 534/1316 (41) |  |
| Combined | 288/1276 (23) | 158/1316 (12) |  |
| Chronic lung disease | 159/1287 (12) | 347/1962 (18) | <0.001 |
| Subvalvular apparatus preservation | 641/802 (80) | 835/1056 (79) | 0.65 |
| Data are presented as N (%) and means ± standard deviations unless specified otherwise. | | | |

**Supplementary data E.** Summary effects for the risk factors for PPM for the following: **(above)** any degree of PPM versus no PPM; **(middle)** moderate PPM versus no PPM; **(below)** severe PPM versus no PPM.

| Any PPM | | | | | | | | | |
| --- | --- | --- | --- | --- | --- | --- | --- | --- | --- |
|  |  |  | Fixed effects model | | |  | Random effects model | | |
| Risk factor | Number of studies | Number of patients | Odds ratio/ mean difference | 95% confidence interval | P-value |  | Odds ratio/ mean difference | 95% confidence interval | P-value |
| Age | 19 | 11675 | 2.80 | 2.32-3.29 | <0.001 |  | 1.21 | -0.66-3.08 | 0.20 |
| Gender (female) | 18 | 10910 | 0.55 | 0.50-0.61 | <0.001 |  | 0.57 | 0.41-0.78 | <0.001 |
| Hypertension | 12 | 5798 | 1.26 | 1.10-1.46 | 0.001 |  | 1.28 | 1.03-1.60 | 0.03 |
| Pulmonary hypertension | 5 | 3833 | 1.21 | 1.02-1.44 | 0.02 |  | 1.21 | 1.02-1.44 | 0.03 |
| Diabetes mellitus | 13 | 6564 | 1.71 | 1.46-2.01 | <0.001 |  | 1.63 | 1.31-2.03 | <0.001 |
| Chronic renal disease | 7 | 3897 | 1.70 | 1.30-2.23 | <0.001 |  | 1.69 | 1.28-2.22 | <0.001 |
| Atrial fibrillation | 14 | 9203 | 0.81 | 0.73-0.89 | <0.001 |  | 0.83 | 0.65-1.07 | 0.14 |
| Impaired LV function | 5 | 5296 | 1.07 | 0.91-1.26 | 0.39 |  | 1.00 | 0.70-1.43 | 0.99 |
| Bioprosthesis | 15 | 9253 | 2.61 | 2.34-2.90 | <0.001 |  | 2.89 | 1.86-4.50 | <0.001 |
| Moderate PPM | | | | | | | | | |
| Age | 6 | 5140 | 0.51 | -0.29-1.31 | 0.22 |  | 0.51 | -0.83-1.85 | 0.46 |
| Gender (female) | 5 | 4482 | 0.45 | 0.39-0.52 | <0.001 |  | 0.45 | 0.26-0.80 | 0.007 |
| Hypertension | 3 | 2341 | 1.15 | 0.95-1.41 | 0.16 |  | 1.19 | 0.84-1.68 | 0.34 |
| Pulmonary hypertension | 2 | 2577 | 1.12 | 0.92-1.37 | 0.26 |  | 1.12 | 0.92-1.37 | 0.26 |
| Diabetes mellitus | 4 | 2999 | 1.89 | 1.51-2.37 | <0.001 |  | 1.92 | 1.44-2.55 | <0.001 |
| Chronic renal disease | 3 | 2379 | 1.57 | 1.16-2.12 | 0.003 |  | 1.64 | 0.97-2.75 | 0.06 |
| Atrial fibrillation | 5 | 4482 | 0.77 | 0.67-0.88 | <0.001 |  | 0.77 | 0.64+-0.92 | 0.005 |
| Impaired LV function | 2 | 2805 | 1.33 | 1.08-1.63 | 0.007 |  | 1.33 | 1.08-1.63 | 0.007 |
| Bioprosthesis | 5 | 4520 | 1.59 | 1.37-1.84 | <0.001 |  | 1.72 | 1.01-2.93 | 0.05 |
| Severe PPM | | | | | | | | | |
| Age | 6 | 2486 | 1.22 | 0.01-2.44 | 0.05 |  | 2.07 | -1.00-5.14 | 0.19 |
| Gender (female) | 5 | 2007 | 0.34 | 0.28-0.42 | <0.001 |  | 0.44 | 0.16-1.21 | 0.11 |
| Hypertension | 3 | 1187 | 1.50 | 1.10-2.05 | 0.01 |  | 1.43 | 0.79-2.60 | 0.24 |
| Pulmonary hypertension | 2 | 1133 | 2.06 | 1.60-2.66 | <0.001 |  | 1.82 | 0.93-3.58 | 0.08 |
| Diabetes mellitus | 4 | 1656 | 3.12 | 2.33-4.16 | <0.001 |  | 3.12 | 2.30-4.22 | <0.001 |
| Chronic renal disease | 3 | 1238 | 2.41 | 1.63-3.55 | <0.001 |  | 2.40 | 1.63-3.56 | <0.001 |
| Atrial fibrillation | 5 | 2007 | 0.52 | 0.43-0.64 | <0.001 |  | 0.57 | 0.36-0.88 | 0.01 |
| Impaired LV function | 2 | 1148 | 1.90 | 1.47-2.46 | <0.001 |  | 1.67 | 0.60-4.70 | 0.33 |
| Bioprosthesis | 5 | 2058 | 2.94 | 2.42-3.58 | <0.001 |  | 6.77 | 1.90-24.08 | 0.003 |

**Supplementary data F.** Funnel plot analysis for early mortality.


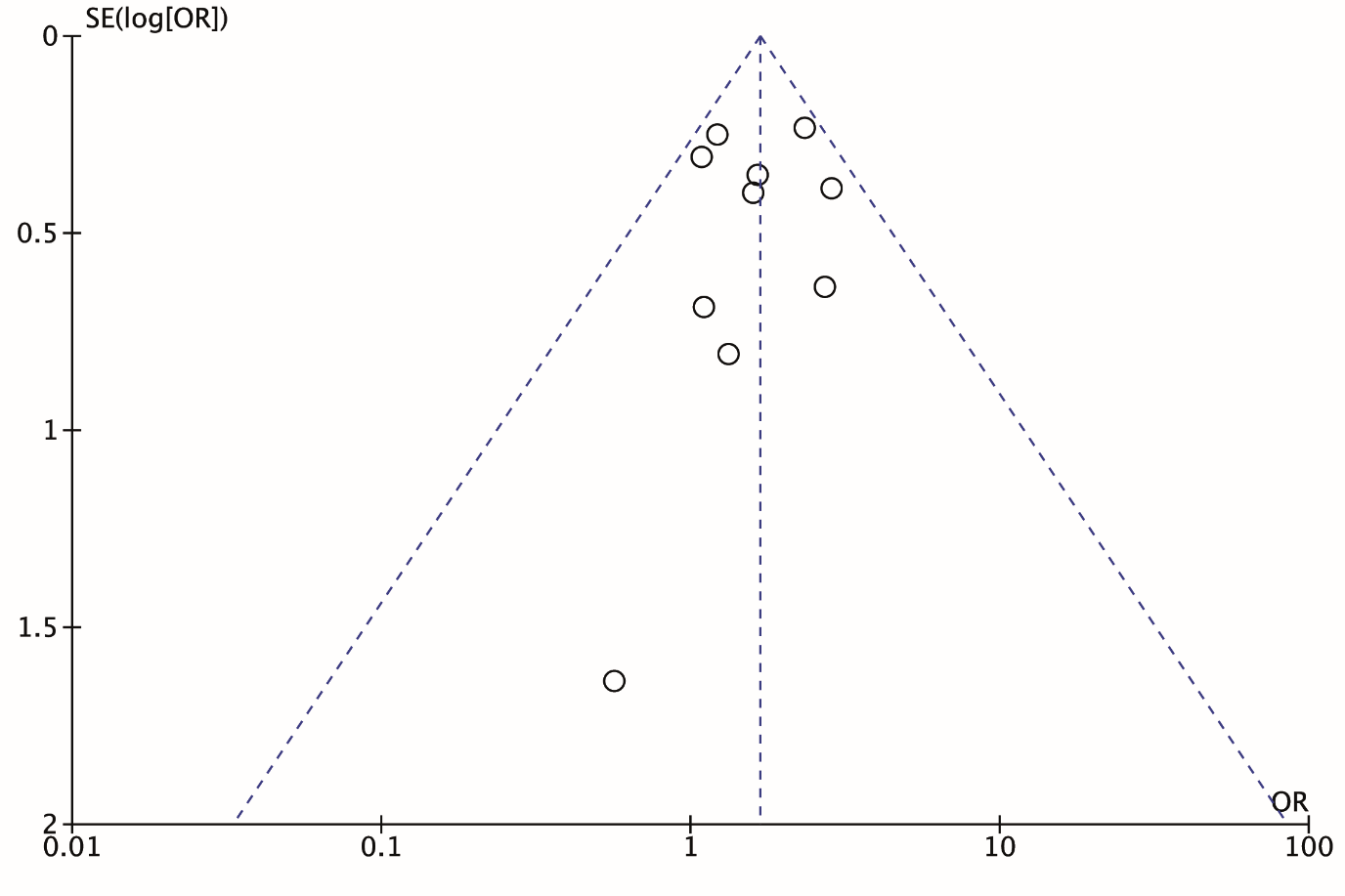


**Supplementary data G.** Subgroup analyses to explore the source of heterogeneity: perioperative mortality **(above)** and overall survival **(below)** were analysed according to baseline- and study-related factors.

**
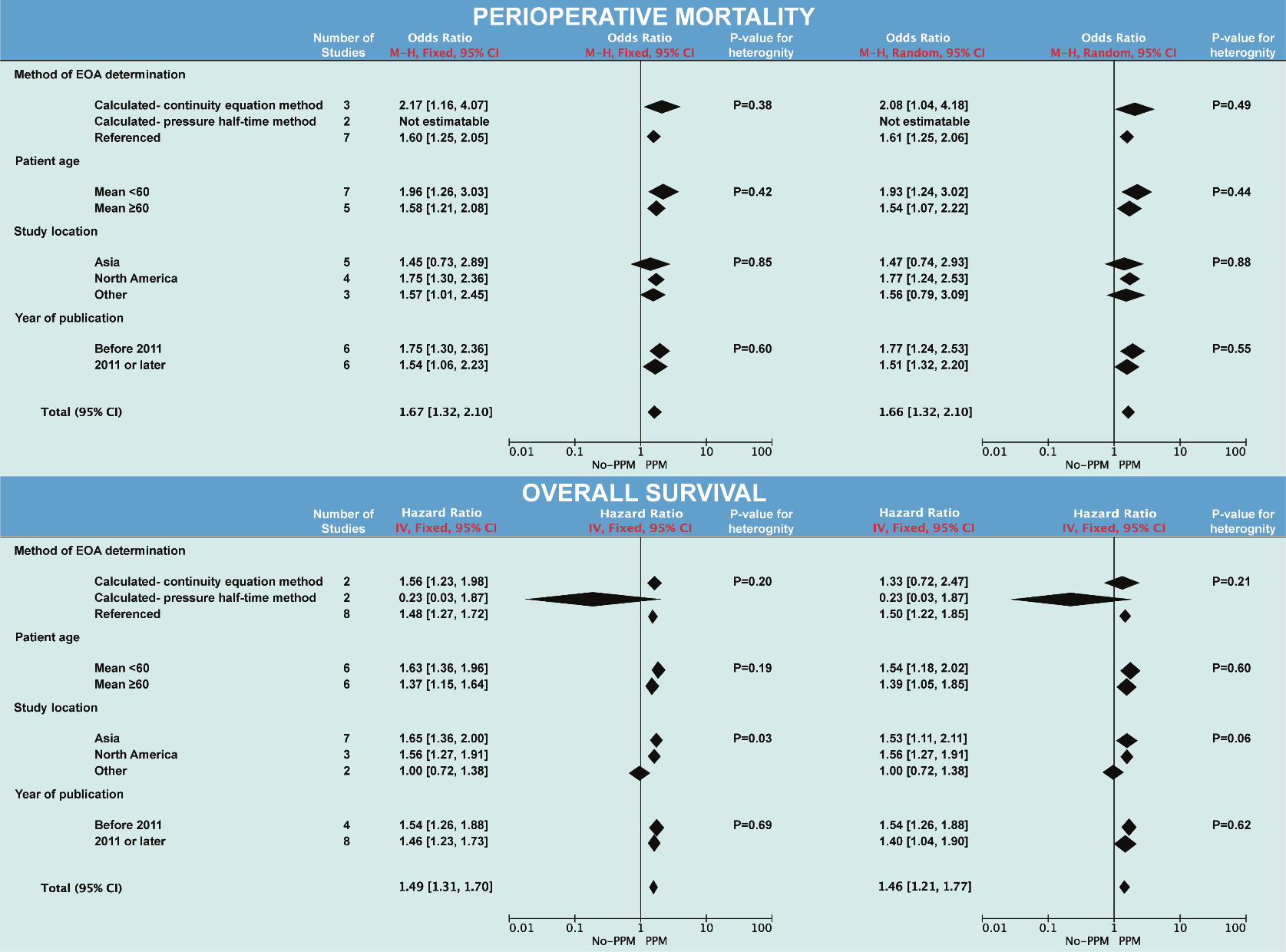
**

**Supplementary data H.** Funnel plot analysis for late overall survival for all studies (above) and after exclusion of studies in which the prosthetic effective orifice area was determined by the pressure half-time method (below).


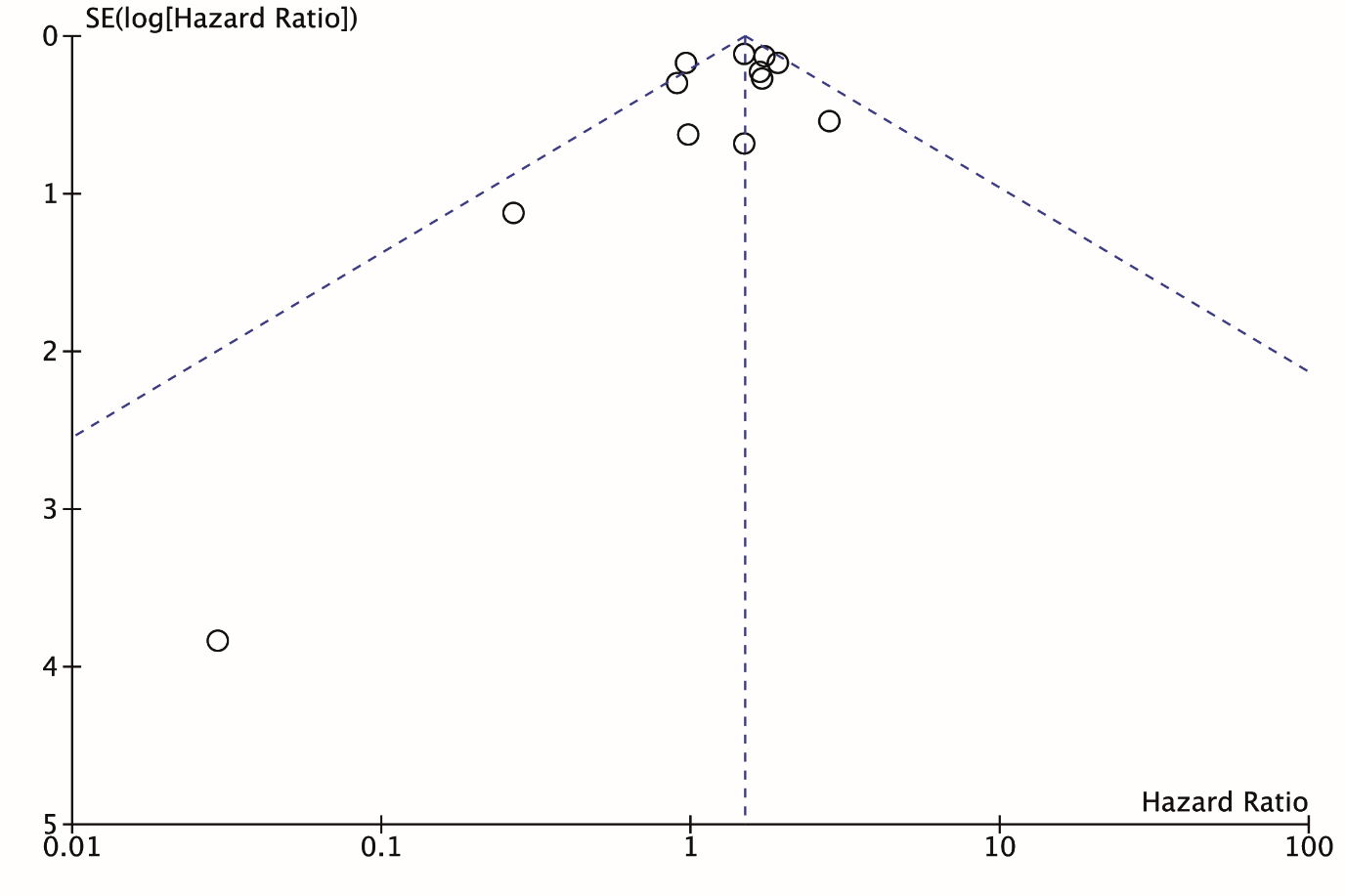


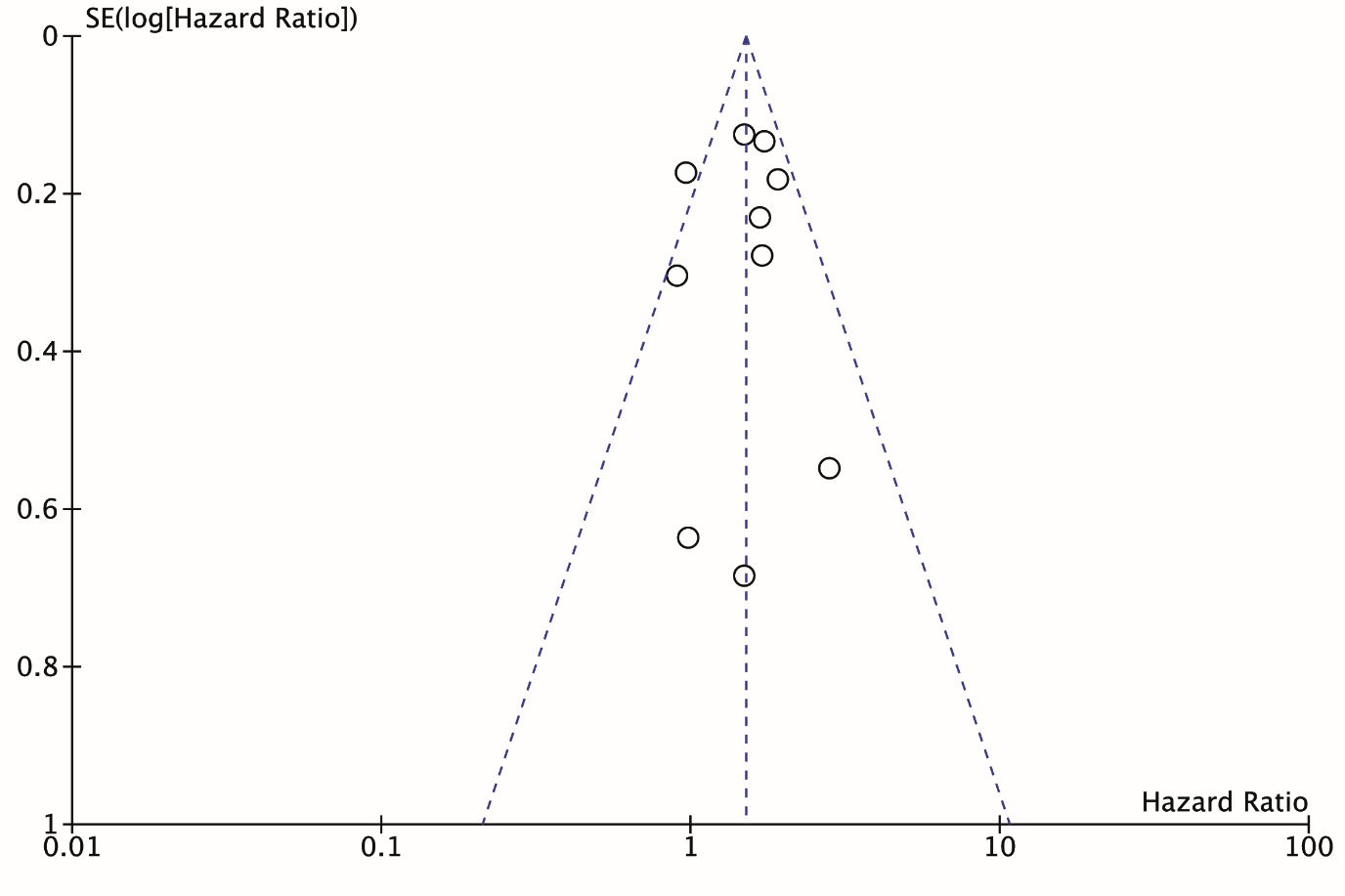


**Supplementary data I.** Forest plot analyses for secondary outcomes.


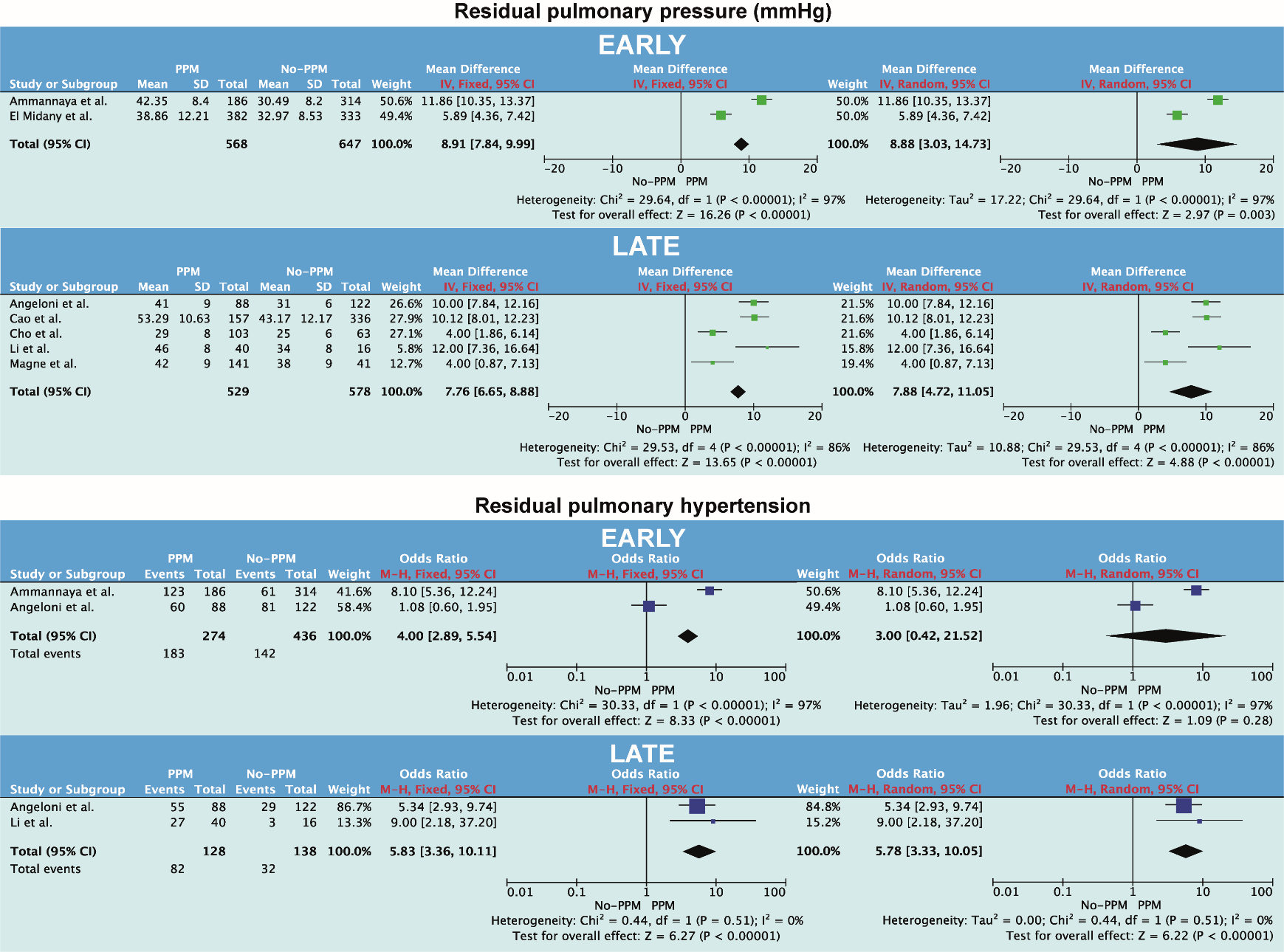


**Supplementary data J.** Results of the univariable meta-regression analysis on the effect of clinical relevant factors on overall survival.

|  | Number of studies | B coefficient | Standard error | P-value |
| --- | --- | --- | --- | --- |
| Age | 12 | -0.011 | 0.008 | 0.20 |
| Gender (female) | 12 | 0.23 | 0.007 | 0.005 |
| Atrial fibrillation | 11 | 0.006 | 0.005 | 0.33 |
| Hypertension | 8 | -0.013 | 0.005 | 0.041 |
| Diabetes mellitus | 8 | -0.013 | 0.035 | 0.73 |
| Impaired left ventricular function | 6 | -0.017 | 0.002 | 0.002 |
